# ‘It might help, but it won’t fix me’: a qualitative study of individuals’ beliefs about manual therapy for low back pain

**DOI:** 10.1186/s12998-026-00647-x

**Published:** 2026-05-14

**Authors:** Mark Thomas, Oliver P. Thomson, Daniel C. Kolubinski, Adele Stewart-Lord

**Affiliations:** 1 London South Bank University, 103 Borough Road, SE1 0AA London, UK; 2 Centre for Osteopathic Research and Leadership (CORaL), Health Sciences University, 275 Borough High Street, SE1 1JE London, UK

**Keywords:** Manual therapy, Low back pain, Health beliefs, Patient safety, Self-management, Therapeutic alliance, Qualitative research

## Abstract

**Background:**

Manual therapy is recommended for the management of low back pain (LBP). However, there are gaps in our knowledge about how individuals understand and view the role of manual therapy in the management of their LBP. Beliefs about manual therapy may influence behaviors and clinical outcomes. This study aimed to develop an in-depth understanding of the beliefs about manual therapy held by a group of individuals experiencing LBP.

**Methods:**

Twenty-one participants with a history of LBP and varied experience of manual therapy participated in semi-structured interviews. The interviews were transcribed verbatim, and the dataset was analyzed using reflexive thematic analysis. A patient and public involvement group contributed to the design of the study.

**Results:**

Five themes were generated: (1) Manual therapy as movement. Manual therapy is predominantly believed to be a transient mechanical treatment aimed at addressing restriction in the back. (2) Self-management and manual therapy go ‘hand in hand’. Self-management was seen as a fundamental and complementary element of manual therapy. (3) Helping me manage or trying to ‘fix’ my back. The participants who believed that manual therapy was an appropriate long-term treatment felt that it could help manage their LBP rather than provide a permanent ‘fix’. (4) All or nothing: the value of the therapeutic alliance. Beliefs about the importance of the therapeutic alliance with a manual therapist varied considerably, with some participants being dismissive of its value. (5) In safe hands? Most participants had no concerns about the safety of manual therapy, conditional on their trust in the manual therapist. However, some participants expressed significant concerns about safety.

**Conclusion:**

Participants’ beliefs about their LBP and the role of manual therapy were shaped by a predominantly mechanical model. However, participants with greater experience of manual therapy often perceived the therapeutic alliance as beneficial for managing their LBP and addressing psychosocial factors. Manual therapists should be aware of unhelpful beliefs held by LBP patients that can potentially influence behaviors and outcomes. In addition, patients’ beliefs about the risks and benefits of treatment should be explored to facilitate shared decision making around the use of manual therapy.

**Supplementary Information:**

The online version contains supplementary material available at 10.1186/s12998-026-00647-x.

## Background

Low back pain (LBP) is a complex condition that is currently the leading cause of years lived with disability globally [1]. There are numerous management options available for individuals experiencing LBP, including manual therapy. Manual therapy includes the modalities of massage, mobilization and manipulation and is utilized by several regulated healthcare professionals. In a contemporary and integrated format, it is a complex intervention within a patient-centered interaction, fostering patient engagement in activity [2]. Manual therapy is considered safe and effective for a number of musculoskeletal conditions, including LBP [3, 4], and is recommended within national and international guidelines [5, 6]. However, as a passive modality, it is recommended as a second-line intervention to be used as an adjunct to education and self-management [7].

According to the common-sense model of self-regulation (CSM), when faced with medical symptoms, an individual will form beliefs about the illness as well as associated beliefs about treatment [8]. Beliefs about an illness have been demonstrated to be important within healthcare, as they can influence patient behaviors and clinical outcomes [9–11]. This relationship has been demonstrated within LBP populations [12–14]. Patient beliefs about LBP are therefore important modifiable targets for clinicians to address [15].

Within the healthcare literature, the focus has been on illness beliefs; however, treatment beliefs have also been investigated. In particular, beliefs about treatment necessity and concerns are significantly associated with medication adherence across a range of physical and psychological medical symptoms [16]. Within the LBP population, treatment beliefs are predictive of adherence to acupuncture appointments [17].

Our previous scoping review mapped the literature on attitudes and beliefs about manual therapy in individuals experiencing LBP [18]. Among the included studies, patients frequently preferred manual therapy, which they considered safe and effective. However, several gaps in our current understanding have been identified, including individuals’ beliefs about the mechanism of manual therapy, the long-term effectiveness of manual therapy, and the relationship between self-management and manual therapy [18]. Treatment beliefs about manual therapy in the management of LBP are important to explore further owing to the current gap in our understanding and their potential to influence behaviors and outcomes. Furthermore, the understanding of contextual factors, which include patient beliefs, has been identified as a research gap and priority for further study within manual therapy [19]. The aim of this study is to develop an in-depth understanding of the beliefs about manual therapy held by a group of individuals experiencing LBP.

## Methods

### Study design

A qualitative study design was utilized, and semi-structured interviews were conducted with 21 participants with experience with LBP. The study was designed, conducted, and reported in accordance with the Consolidated Criteria for Reporting Qualitative Research (COREQ) checklist [20]. An interview guide (Additional file 1) was developed from our previous scoping review [18], the CSM [8] and the clinical experience of the research team. Reflexive thematic analysis (RTA) was used to analyze the data, taking an experiential and critical realist position [21]. This provides a suitable approach that is compatible with the research aims and study design, namely, exploring participant experiences and the sense-making of a phenomenon across a sample population [22].

## Patient and public involvement

A patient and public involvement (PPI) group, consisting of three individuals with lived experience of LBP, was integral to the design of the study. They reviewed all the participant-facing information, ensuring the clarity and accessibility of the wording. In addition, they participated in pilot interviews, leading to a clearer and more meaningful interview guide (Additional file 1).

## Participants

Participants were eligible if they were aged 18 years or over and were currently experiencing, or had a history of, recurring non-specific LBP (with or without leg pain) of any duration. Participants were excluded if they were students or qualified manual therapists or if their LBP was related to pregnancy or previous spinal surgery. All 21 participants consented and participated in semi-structured interviews conducted by the primary researcher (MT). To ensure confidentiality, all participants were offered the opportunity to choose a pseudonym; alternatively, a pseudonym was randomly assigned [23].

## Recruitment

Recruitment took place through multiple sources across the London region, including London South Bank University campuses, local manual therapy clinics, university teaching clinics (e.g., the University College of Osteopathy), the Croydon Black and Minority Ethnic forum, and social media platforms. A broad recruitment strategy was used to obtain variation in participant demographics, the duration of LBP, and previous experience of manual therapy. Interested individuals completed eligibility screening and provided contact details.

There were 56 expressions of interest, of which 48 individuals met the eligibility criteria. Eligible individuals were contacted via email, and invitations were issued on a first-come basis. Twenty-seven individuals did not respond, declined, or replied outside of the recruitment window. In total, 21 participants agreed to participate and provided written consent.

Consistent with the authors of RTA, data saturation was not used due to its limitations and incompatibility with RTA [24]. Instead, guided by the concept of information power, a sample of approximately 20 participants was deemed appropriate to achieve richness of data and variability in the sample to address the research aims [25].

## Data collection

The interviews were conducted between the researcher and each participant between January and March 2024, either online via Microsoft Teams (*n* = 16) or within a private setting, e.g., clinical space (*n* = 5), based on the preference of the participant. The duration of the interviews ranged from 29 to 56 min. No repeat interviews occurred. All interviews were recorded via Microsoft Teams, with field notes made during and after the interviews. Initial transcription was automated via Microsoft Teams. The primary researcher (MT) checked all transcripts to ensure accuracy and to gain familiarity with the data. The transcripts were returned to all the participants for review, with the option of providing corrections or additional comments [26].

### Research team and reflexivity

The primary researcher (MT) is a PhD student and educator with previous experience in manual therapy as a chiropractor and physiotherapist. He received additional training in designing and conducting qualitative interviews. A reflexive journal was kept prior to, during, and following the study design, data collection, and analysis [21]. The research team also comprised a radiographer and mixed-methods researcher (ASL), an osteopath and qualitative researcher (OT), and a psychologist and quantitative researcher (DK).

### Data analysis

RTA was utilized to generate themes within the data via NVivo qualitative data analysis software [27]. This process involved becoming familiar with the dataset and initial coding, followed by generating, developing, refining, and naming themes [21]. MT generated and labelled the initial codes inductively within the dataset. MT and ASL reviewed and discussed the developing codes through ongoing discussion. MT and ASL generated the candidate themes by clustering codes reflecting shared patterns of meaning. The wider research team (MT, ASL, OT, and DK) further developed, refined and named the themes through ongoing discussion.

## Results

The participant demographics for the sample are presented in Table 1, and individual participants’ experiences with LBP and manual therapy are shown in Table 2.

**Table 1:** Participant group demographics (*N* = 21)

Demographic characteristic	*n* (%)
**Gender**	
Female	9 (42.9)
Male	12 (57.1)
**Age**	
18 to 24	2 (9.5)
25 to 34	1 (4.8)
35 to 44	6 (28.6)
45 to 54	7 (33.3)
55 to 64	2 (9.5)
65 to 74	2 (9.5)
Not disclosed	1 (4.8)
**Ethnic group**	
Asian or Asian British	2 (9.5)
Black, Black British, Caribbean or African	4 (19.1)
Mixed or multiple ethnic group	2 (9.5)
White	11 (52.4)
Other ethnic group	1 (4.8)
Not disclosed	1 (4.8)
**Employment status**	
Employed or self-employed	16 (76.2)
Student	3 (14.3)
Retired	1 (4.8)
Not disclosed	1 (4.8)

**Table 2:** Participant experience with LBP and manual therapy

Pseudonym	LBP duration	Healthcare professionals consulted for LBP	Modality of manual therapy experienced	Current or previous manual therapy experience
Allen	5 months	GP, Osteo	Manipulation, massage	Short-term treatment (e.g. a few sessions)
Arron	5 years	Chiro, GP, Physio, Osteo, Surgeon	Manipulation, massage	Ongoing or regular treatment
Augustus	10 years	Chiro, Massage, Physio, Osteo	Manipulation, massage, mobilization	Ongoing or regular treatment
Bradley	3 years	GP, Physio	None	No experience
Brendon	30 years	GP, Physio, Surgeon	Manipulation, massage, mobilization	Treatment on multiple occasions over time
Carol	20 years	Chiro, Massage, Physio, Osteo	Manipulation, massage	Ongoing or regular treatment
Corey	5 years	GP	None	No experience
Elisabeth	10 years	None	None	No experience
Elsie	7 years	GP, Physio	None	No experience
Florence	40 years	Chiro, GP, Physio	Manipulation	Ongoing or regular treatment
Gertrude	3 years	TCM	None	No experience
Jamison	6 years	Chiro, GP	Manipulation, massage	Short-term treatment (e.g. a few sessions)
Majorie	30 years	Chiro, Massage, Osteo	Manipulation, massage	Ongoing or regular treatment
Martin	20 years	Chiro, Massage, Physio, Osteo	Manipulation, massage, mobilization	Treatment on multiple occasions over time
Nathan	10 years	GP, Physio, Osteo	Manipulation, massage	Treatment on multiple occasions over time
Noel	26 years	GP, Physio, Sport	Manipulation, massage	Short-term treatment (e.g. a few sessions)
Penelope	10 years	GP, Physio, TCM	Massage	Ongoing or regular treatment
Sterling	3 weeks	Chiro, GP, TCM	Manipulation, massage	Short-term treatment (e.g. a few sessions)
Tania	9 years	Physio	Massage	Short-term treatment (e.g. a few sessions)
Valeria	20 years	Chiro, Massage	Manipulation, massage	Ongoing or regular treatment
Wesley	40 years	Chiro, GP, Physio	Manipulation, massage	Short-term treatment (e.g. a few sessions)

Key: Chiro = chiropractor; GP = general practitioner; Massage = massage therapist; Physio = physiotherapist; Osteo = osteopath; Sport = sport rehabilitator; TCM = traditional Chinese medicine practitioner.

The data analysis resulted in the generation of five themes that describe the range of beliefs held by participants:

### Theme 1: Manual therapy as movement

The participants held dominant underlying beliefs about the mechanical nature of LBP. These mechanical causes of LBP include tightness or weakness in the back, (poor) posture, (incorrect) lifting and carrying, age, and increased body weight. Tightness in the back was commonly described as a causal factor and therefore an appropriate target for treatment. Manual therapy was perceived to address this mechanical restriction by creating movement in the spine:

*“I just thought it [my back] was very*,* very rigid*,* and they’re trying to get some flexibility into it. The whole thing [manual therapy] was focused on getting the back moving a bit more flexibly.”* (Brendon).

With the mechanical force applied during treatment as the mechanism of action, the effect of manual therapy was perceived to be transient in nature. Most participants believed that manual therapy would result in short-term pain reduction and improved function, ranging from a few days to several weeks:

*“And then later that day it [my back] is free and the next day it’s freer. And then it kind of slowly*,* kind of tightens up*,* stiffens up.”* (Arron).

In further exploration of beliefs about the mechanism of manual therapy, most participants expressed significant uncertainty in relation to the anatomical structures involved or the exact mechanism of action:

*“I mean*,* is it [manual therapy] to do with the muscles or is it to do with the bones? I’m not quite sure actually.”* (Elisabeth).

*“I mean I’m no spine expert […] It’s not your spine*,* cracking*,* it’s the fluid that’s releasing in between each vertebra. But*,* I don’t know if that is right? Is that wrong? I have no idea.”* (Noel).

In general, participants viewed manual therapy as working through movement; however, many appeared more concerned with its effectiveness rather than the exact mechanism of action:

*“I don’t need to know how it [manual therapy] works. I mean I’m more interested*,* to make sure that it works.”* (Bradley).

### Theme 2: Self-management and manual therapy go ‘hand in hand’

Self-management was viewed as a fundamental part of LBP management by all participants. Most participants believed the key element of self-management to be exercise, facilitating movement through the spine, normally to improve flexibility and/or strengthen the back:

*“I think I’ve been wound up like a tennis ball for years*,* through not actually exercising and not doing any core work as well. And core work I think*,* essentially would stabilize my back. So*,* I need to focus on that a little bit more*,* but yeah*,* stretching has definitely helped and just being active has helped.”* (Noel).

A few participants viewed exercise and manual therapy on a continuum, with both generating movement to address restriction in the spine. However, manual therapy was able to create greater and more specific movements than exercise:

*“It [manual therapy] targets the areas of your body that you normally don’t expect that sensation at. The stretches that I know*,* I know how they would feel*,* but when I go to manual therapy*,* I feel stretches in a very new place”.* (Allen)

Most other participants viewed self-management and manual therapy as complementary but distinct approaches. Self-management was seen to modify wider lifestyle choices and behaviors but was still predominantly focused on addressing mechanical causes of LBP, such as (poor) posture:

*“It’s important for the patient to actually have that self-management*,* because without the patient doing bits with regards to nutrition*,* you know being mindful of their posture*,* doing relevant*,* you know*,* strengthening exercises*,* then the actual manual therapy will be less effective for that person in that pain management*,* that it*,* kind of it goes*,* hand in hand.”* (Valeria).

Many participants valued the role of a manual therapist in supporting self-management, providing encouragement and guidance for an approach that requires motivation and resilience:

*“I think self-management is a really important discipline and self-management’s hard to maintain*,* going to a [manual] therapist kind of helps a bit with maintaining some sort of rhythm.”* (Augustus).

Overall, all the participants were confident that manual therapy and self-management were compatible and that this was a common-sense approach. This belief was based on both manual therapy and self-management primarily looking to address mechanical dysfunction in the back. No participant held contradictory beliefs, such as that the approaches were incompatible or that manual therapy did not require self-management:

*“They’re hand in hand*,* aren’t they? Yeah*,* I see it that they are hand in hand*,* aren’t they? Well*,* it’s logical*,* I’m quite logical. One must go with the other.”* (Arron).

### Theme 3: Helping me manage or ‘fixing’ my back

For several participants, manual therapy was viewed as the appropriate treatment for the ongoing management of their LBP. These participants often had greater experience of manual therapy, including engaging in and benefiting from regular care:

*“I actually think I’m doing the right thing*,* I think I found the right treatment […] I don’t really believe in doing anything like going to the GP […] but I feel like an osteopath or a massage is trying to make it better*,* but for the long-term.*” (Majorie).

The participants who regarded manual therapy as beneficial over the long term were comfortable with the temporary effect of manual therapy and often believed in the concept of managing their LBP symptoms rather than finding a ‘fix’. They were accepting of the persistent or recurring nature of LBP and were looking for ways to control their pain experience:

*“It [low back pain] is a condition that can ease off but will probably never get better. So*,* it’s just controlling it.”* (Noel).

In contrast to the positive views about manual therapy, several participants did not regard it as an appropriate treatment for their LBP because of a lack of effectiveness:

*“Chiropractic treatment had little or no effect. Yeah*,* I admit*,* it didn’t make it worse […] I didn’t really see any marked benefit from it.”* (Wesley).

Many of these participants were often focused on the requirement to ‘fix’ the mechanical problem in their low back, i.e., have a long-term or permanent solution to completely resolve their LBP:

*“Maybe I need some sort of operation to like, to fix the joints I think*,* because surely after massage*,* I should be right after a while.”* (Nathan).

However, the ‘fix’ often remained elusive, resulting in frustration and dissatisfaction with the attempted treatment approaches for some of these participants.

*“Why can’t I fix it [my low back pain]? Why doesn’t it get better?”* (Arron).

*So why is it [my low back pain] not getting resolved? […] why isn’t the medical world coming up with a solution that works for everyone?* (Bradley)

### Theme 4: All or nothing; the value of the therapeutic alliance

The participants held varying views on the importance of the therapeutic alliance between the manual therapist and the patient, ranging from regarding it as unnecessary to considering it essential.

The participants who saw little value in this relationship often had limited experience of manual therapy and held beliefs about the need to “fix” the back. For these participants, the value of treatment was solely the technical expertise of the manual therapist rather than the interpersonal interaction:

*“They [the manual therapist] have got the skills and their experience and the knowledge*,* I don’t think it should matter who it is.”* (Tania).

*“Say if there is a problem and I know how to solve it*,* that’s what matters. I don’t think personal relationships help.”* (Bradley).

Some accepted that a positive relationship could be beneficial but not essential. However, this relationship was often seen as temporary and performative rather than meaningful and lasting:

*“They don’t have to be the friendliest person in the world*,* but I think it’s nicer if you feel a little bit of like warmth towards that person.”* (Gertrude).

For a few participants who did not see value in the therapeutic alliance, any dialog or rapport was only seen as a customary pleasantry during treatment, akin to any other transactional service:

*“it’s just you’re getting a service [with Osteopathy]*,* could be a person doing your coffee or your cab driver […] I wouldn’t mind if it was done in complete silence […] quite simply*,* I didn’t come here for a chat.”* (Allen).

In contrast, many other participants, particularly those with greater experience of manual therapy and who regarded it as a beneficial way to manage their LBP, described a closer and more meaningful therapeutic alliance. For these participants, the relationship with the manual therapist was paramount and an active component of their positive outcomes. They valued being truly respected and heard, with their LBP experience being validated:

*“I think it is vital to [have a] very good relationship between you and that person [the manual therapist] […] I was treated with respect […] Understanding*,* knowledgeable*,* attentive and just a good listener.”* (Jamison).

Many participants valued care being individualized to them with the manual therapist able to demonstrate empathy and genuine interest:

*“They [the manual therapist] know you*,* and they’re interested. Opposed to oh*,* you’re just another back on the slab*.” (Brendon).

Some participants also described the comfort and reassurance associated with someone caring for them and supporting them with their LBP experience on an ongoing or regular basis:

*“So*,* there’s definitely something when you’re being looked after on a regular basis […] I think there’s also the mental effect of things*,* that you’ve got someone who’s looking after you*,* and who understands. And mentally you feel in a better place.”* (Marjorie).

Despite holding underlying mechanical beliefs, many of these participants appeared to have a more holistic view of their LBP, with beliefs about the complexity of pain, including the role of psychosocial factors such as stress, mood and cognition. These participants felt the interaction with the manual therapist could address some of the psychosocial factors they associated with their LBP:

*“90% of illness is caused by stress*,* aren’t they? So*,* you know*,* stress is very important. Stress can cause a lot of illnesses*,* back pains*,* muscle problems […] my chiropractor always has to check on me*,* to make sure I haven’t had a stressful day.”* (Valeria).

### Theme 5: In safe hands?

Most participants with experience of manual therapy expected and accepted some degree of discomfort, which they justified by the perceived benefits of treatment:

*It [manual therapy] is kind of painful*,* but*,* actually when I come out*,* I feel much better.”* (Martin).

However, some participants who were naïve to manual therapy felt that the anticipated pain could be a barrier to them receiving care, regardless of the potential benefit:

*“But apparently it [manual therapy] works really well. You know*,* so that’s one positive*,* although I don’t know if I want to be put through that amount of pain.”* (Elisabeth).

Other than potential discomfort, many participants had no concerns about receiving manual therapy. In fact, participants were often very certain in their attitudes toward manual therapy safety:

*“If I had any concerns*,* I wouldn’t have gone […] I had 100% faith and trust in him [the manual therapist] completely.”* (Florence).

With these participants, the lack of concern was fundamentally conditional on the trust they had in the manual therapist:

*“I mean [I am] completely immersed in trust*,* in what they [the manual therapist] are doing […] I just immerse myself*,* fully trust them and just let them do their thing. So of course*,* it’s full trust in the people that I see*,* definitely.”* (Arron).

Conversely, in some participants, clear concerns about manual therapy did exist, normally owing to a fear that they could become worse rather than better:

*“I just think that I’m scared that it [manual therapy] will bring back the pain”.* (Penelope)

There were three key reasons for safety concerns, each linked to a different set of participant beliefs: (1) beliefs about their own LBP, (2) beliefs about the characteristics of the manual therapist, and (3) beliefs about the manual therapy technique. The first reason, present in a few participants, centered on an underlying belief that the spine was a susceptible and delicate structure; therefore, manual therapy could result in harm:

*“I’ve always been concerned about anyone touching my back*,* to be honest*,* but in fear that it might trigger something worse*,* or in fact*,* I always thought that my spine was maybe a little bit like sawdust*,* after all that it’s been through.”* (Noel).

This concern was occasionally linked to an underlying anxiety that LBP may be a result of sinister pathology. These participants felt that imaging could offer them reassurance prior to manual therapy:

*“Let me take an X-ray first*,* see if it is broken […] he can be massaging it*,* he could be making it worse. You got to see if you got a hairline crack first before you start. That’s my opinion.”* (Nathan).

The second concern, present in several participants, referred to the characteristics of the manual therapist, such as a lack of qualifications and experience:

*“When I researched him [the chiropractor]*,* I know this sounds ridiculous*,* but he was self-taught […] I’m probably doing him an injustice*,* he is probably really good*,* but at the end of the day*,* I wasn’t confident.”* (Florence).

This concern extended to the manual therapist’s age. A couple of participants felt that an older manual therapist would provide gentler and safer care:

*“I probably wouldn’t go to see a 25-year-old male osteopath or chiropractor because I think they probably don’t know their own strength and nothing’s broken on their body yet*,* so they’re less careful with other people’s bodies.”* (Martin).

The final concern, held by a few participants, was based on beliefs about the modality of manual therapy. Massage was universally deemed by all participants to carry little to no threat:

*“But I think for a massage*,* I don’t really get nervous […] Yeah different*,* feels safer. You’re not messing around too much with my body. You’re just*,* yeah*,* you know*,* doing a massage.”* (Gertrude).

However, manipulation, which was normally associated with the professions of chiropractic and osteopathy, carried a greater perceived threat for some participants. This concern was often linked to the ‘cracking sound’ associated with spinal manipulation:

*“Like how chiropractors are seen*,* they are seen as literal backbreakers and it’s like their nickname […] that sound*,* that sound isn’t pleasant […] When you hear that sound*,* you would immediately associate something negative with it.”* (Elisabeth).

## Discussion

Our study explored the beliefs about manual therapy held by individuals experiencing LBP. The findings highlight that individuals hold varying beliefs about manual therapy and the role of the manual therapist in the management of LBP.

The study findings can be interpreted using the common-sense model of self-regulation (CSM) [8]. Participants’ predominantly biomechanical beliefs about LBP reflect illness identity and causation representations within the CSM. These beliefs shaped how participants interpreted both their LBP and the role of manual therapy in its management.

The CSM also supports the findings relating to participants’ beliefs about the value of the therapeutic alliance. From a common-sense perspective, when beliefs about LBP and its treatment are primarily mechanical in nature, it is logical that individuals place greater value on the technical skills of the manual therapist. In contrast, interactive elements of care, such as communication and the therapeutic alliance, may initially be regarded as less relevant. Among participants with greater treatment experience, increased value was placed on the therapeutic alliance, suggesting that treatment beliefs were dynamic rather than static. This finding aligns with the CSM premise that beliefs about illness and treatment are continually appraised and revised on the basis of experience and outcomes [8].

The findings of this study support and extend the application of the CSM to LBP by highlighting the importance of treatment beliefs alongside illness beliefs. As outlined in the introduction, patient beliefs represent a modifiable target for clinicians. Manual therapy encounters therefore provide an opportunity to explore beliefs about LBP, as well as perceived benefits and risks associated with manual therapy.

As stated, the participants held underlying mechanical beliefs about their LBP. It is well established in the literature that individuals experiencing LBP hold these beliefs [28–30]. In addition, these beliefs are prevalent in the general population [31–34] and healthcare professionals, including manual therapists [35].

Previous qualitative studies have described mechanical beliefs in participants regarding the mechanism of manual therapy [36–39]. Our study supports previous findings; however, when the mechanism of action was explored further, most participants held significant uncertainty beyond the concept of producing movement in the back. A recent qualitative study by Petosky et al. [40] directly explored how patients with various musculoskeletal conditions believe that manual therapy works. They also reported that the primary belief was a mechanical action, which predominantly increased mobility. In addition, they identified significant uncertainty in understanding, with participants often defaulting to describing outcomes rather than mechanisms.

A recent study investigated physiotherapists’ beliefs about the mechanism of manual therapy for spinal pain [41]. These beliefs were mainly mechanical and neurophysiological in nature. In addition, the mechanism was believed to be due to a single factor and reflected a limited level of conceptual understanding. It appears that both individuals experiencing LBP and manual therapists hold a more simplified and superficial understanding of the mechanism of manual therapy.

Self-management is recommended as the first-line approach for LBP [7]. The participants in our study were clear about the importance of self-management and how this complemented manual therapy. The participants felt that self-management should be predominantly exercise focused, a finding that has been demonstrated in other qualitative work [42]. For most participants, mechanical beliefs underpinned the strong cohesion between self-management and manual therapy.

Manual therapy has been criticized for its negative relationship with self-management and potential risk of dependence [31, 43]. Our study highlighted that some individuals experiencing LBP perceived benefits from manual therapy interactions to support their self-management. This finding has previously been demonstrated in the literature, with manual therapy considered to support and complement home exercise approaches [44, 45].

There is a concern in the literature that patients can hold unhelpful beliefs about the need to ‘fix’ the mechanical dysfunction they perceive is responsible for their pain [15]. Several participants expressed this view within our study. However, it appeared to be held by participants with no or limited experience of manual therapy, who often held negative beliefs about the effectiveness of manual therapy. This belief was reflected in participants’ accounts of poorer low back pain outcomes and was often accompanied by frustration with the healthcare system and available treatment options.

In contrast, many participants with positive experiences of manual therapy commonly expressed the belief that a ‘fix’ for their LBP was neither possible nor expected. Instead, they viewed LBP as a complex and chronic condition that could be effectively managed with the support of a manual therapist. This included facilitating self-management as well as providing a support structure offering reassurance, empathy, and addressing psychosocial factors. These findings suggest that subgroups of individuals exist that are more likely to benefit from manual therapy. Previous work has highlighted that some patients have varying outcomes from chiropractic maintenance care on the basis of their psychological, behavioral and social characteristics [46, 47].

Within our study, the therapeutic alliance with the manual therapist was key to the perceived long-term benefit of manual therapy. Many participants recognized its value and described developing a strong therapeutic alliance over time. The therapeutic alliance has been demonstrated to be an important contextual factor that positively influences LBP outcomes [48–50] and is an important contributor to patient satisfaction [51]. As such, the therapeutic alliance is considered of paramount importance within contemporary manual therapy [52].

However, our present study highlighted that the therapeutic alliance may not be considered important for some individuals initially engaging in or considering manual therapy. Previous work has demonstrated that, prior to care, individuals experiencing LBP have a stronger preference for manual therapists based on their technical expertise rather than their interpersonal skills [53].

One universally accepted element of the therapeutic alliance was trust when receiving manual therapy. Trust is a multifaceted and complex construct and is fundamental to an effective therapeutic alliance [54]. Within the manual therapy context, greater trust is associated with improved patient outcomes [55]. In addition, trust has been shown to be a critical element in how patients evaluate adverse events (AEs) in manual therapy [56–58].

Owing to their trust in the manual therapist, most of the participants in our study did not have concerns. However, several were concerned about the safety of manual therapy, particularly the risk of aggravating symptoms through mechanical force directed to the lower back. Manual therapy for the management of LBP is a relatively safe treatment. Mild AEs (e.g., post-treatment soreness) are very commonly reported, whereas serious AEs are rare [59, 60]. The participants in our study were accepting of soreness during or after manual therapy, an attitude that has been previously identified in the literature [56, 57].

A few participants were concerned about the vulnerability of their low back; this belief has been previously explored [61] and shown to be prevalent in the population [33]. Some participants feared an underlying pathology, with imaging desired for reassurance. Many individuals experiencing LBP believe that imaging is beneficial [62, 63], despite this being in contradiction to LBP management guidelines [7].

Some participants had specific concerns about manipulation. Like massage and mobilization, manipulation has been demonstrated to be relatively safe in the management of LBP [3, 4]. In addition, manipulation for LBP does not appear to produce different outcomes, including AEs [64]. However, there is certainly an increased perceived risk of serious AEs from manipulation, although this risk is often focused on the cervical spine [65, 66]. In addition, previous qualitative studies have highlighted participants’ sense that manipulation is different from other manual therapy modalities with an increased risk of harm [36, 37].

### Study implications

It is important for manual therapists to address unhelpful beliefs about LBP, such as the vulnerability of the spine or the need for diagnostic imaging. Several validated tools are available to identify and measure these beliefs in individuals experiencing LBP [67, 68]. This is particularly relevant because clinicians have the strongest influence on patients and can inadvertently reinforce unhelpful or inaccurate beliefs about LBP [69].

In addition, clinicians should explore patient beliefs about manual therapy in the management of LBP. To our knowledge, only one validated tool currently measures these beliefs: the LBP Treatment Beliefs Questionnaire (LBP-TBQ) [70], which is used to assess patient preferences for common treatment modalities, including manual therapy.

Biomechanical research has demonstrated credible mechanical changes during manual therapy, particularly temporary changes in the facet joint space and spinal stiffness [71]. However, the current working model for the mechanism of action of manual therapy is complex and multifactorial [72]. Therefore, patient beliefs are not aligned with contemporary understanding. However, explaining the effects of manual therapy is a conceptual challenge, especially in light of such uncertainty. Nonetheless, manual therapists should be clear about limited mechanical effects during treatment, addressing misconceptions and moving away from a traditional mechanical framework [73].

In the literature, there have been calls to modernize the use of manual therapy, integrating hands-on care and empowering individuals to take an active role in the management of their LBP [52, 74, 75]. We advocate this approach and suggest that manual therapy can form an effective package of care that also involves patient education and self-management approaches. Rather than focusing on mechanical dysfunction, self-management should seek to reframe potentially unhelpful beliefs and include problem solving, goal setting, and providing patients with their own tools to independently manage pain [76].

Individuals’ beliefs about the risk of manual therapy may influence their behaviors, for example, engaging in or adhering to manual therapy. In addition, negative beliefs about manual therapy may have a nocebo effect, influencing the experience of AEs [77], which have been reported during sham manual therapy [78]. It is important for manual therapists to identify patient safety concerns to facilitate shared decision making and minimize nocebo effects. Patient beliefs about the risks and benefits of manual therapy should guide treatment decisions, including the modality of manual therapy utilized.

### Strengths and limitations

This study was able to gain rich insights into the beliefs of participants about manual therapy. The recruitment strategy facilitated a diverse range of participant backgrounds and perspectives. This was important, as beliefs about LBP have been shown to differ across sociodemographic groups [32, 79]. Whereas previous work has involved patients currently receiving manual therapy [37, 40], this study included individuals experiencing LBP without previous exposure to manual therapy. This is important, as our study suggests that beliefs about manual therapy may change during clinical encounters. Another strength of this paper was the inclusion of a PPI group in the design of the study, including the interview guide and all participant information. This ensured that the study materials were clear and accessible to the public and that the study design aligned with and considered the patients’ perspective [80].

There were several limitations to this study. First, no validated tool was used to measure the disability and pain levels experienced by participants. This is important, as higher disability and pain levels have been associated with negative beliefs about LBP [81]. Second, the participants’ LBP duration in this study was mainly limited to chronic symptoms. Although individuals experiencing acute and subacute symptoms were eligible, this population is more challenging to recruit as research participants [82]. Therefore, the findings might not be transferable to individuals experiencing acute LBP, who can hold different beliefs from individuals experiencing chronic LBP [83].

Third, the study was conducted in the United Kingdom (UK), which has a unique healthcare model with clear differentiation between public and private healthcare. The participants’ experience of manual therapy in this study was almost exclusively in private settings rather than in the public system, the National Health Service (NHS). Alternative healthcare systems in other countries may influence the beliefs about manual therapy held by individuals experiencing LBP.

Fourth, as participants expressed significant uncertainty around the mechanism of manual therapy, it was challenging to elicit meaningful beliefs from participants, both from individuals with and without experience of manual therapy. Finally, as the interviews were conducted by a trained manual therapist, this may have influenced participants’ responses, resulting in increased willingness to discuss the benefits rather than the risks of treatment.

## Conclusion

Manual therapy was predominantly perceived as a transient, mechanical treatment aimed at addressing restriction in the back; however, participants expressed significant uncertainty regarding its mechanism of action. Some participants, particularly those with greater experience of manual therapy, also conceptualized it through a biopsychosocial lens.

Self-management was seen as a fundamental and complementary element of care, described as working ‘hand in hand’ with manual therapy. Participants who viewed manual therapy as an appropriate long-term treatment believed that it could help manage their LBP rather than provide a permanent ‘fix’.

Participants’ beliefs about the importance of their therapeutic alliance with the manual therapist varied considerably, with some participants placing limited value on the relationship. Most participants reported no concerns about the safety of manual therapy, conditional on their trust in the manual therapist. However, some participants expressed significant safety concerns, perceiving a potential risk of harm associated with manual therapy.

Communicating the role of manual therapy presents a clinical challenge for manual therapists due to the complexity and uncertainty involved. As a key influence on patient beliefs, manual therapists must ensure that patients are not encouraged to establish unhelpful beliefs that could negatively impact their behaviors and outcomes. Exploring patients’ beliefs about the risks and benefits of manual therapy is important to support patients in making informed decisions about their care.

## Supplementary Information

Below is the link to the electronic supplementary material.

Supplementary Material 1

## Data Availability

The datasets generated and analyzed during this study consisted of qualitative interview transcripts. Owing to the sensitive and confidential nature of the data, the full transcripts cannot be shared publicly to protect participant privacy. However, anonymised excerpts relevant to the findings are available from the corresponding author upon reasonable request and subject to ethical approval.

## Abbreviations

AE: Adverse event
CSM: Common-sense model of self-regulation
GP: General practitioner
LBP: Low back pain
LBP‑TBQ: Low Back Pain Treatment Beliefs Questionnaire
NHS: National Health Service
PPI: Patient and public involvement
RTA: Reflexive thematic analysis
UK: United Kingdom
