# Supplementary material for: ‘It might help, but it won’t fix me’: a qualitative study of individuals’ beliefs about manual therapy for low back pain
Source: Chiropr Man Therap. 2026 May 14;34:20. doi: 10.1186/s12998-026-00647-x (PMC13191850; doi:10.1186/s12998-026-00647-x)
Supplement: Supplementary file 1 — Supplementary Material 1 [file 12998_2026_647_MOESM1_ESM.pdf]

Additional file 1: Interview Guide

"Thank you for agreeing to take part in this interview. Please can you confirm you have read the participant information sheet and are happy to go ahead? Do you have any questions before we start?"

"My name is Mark Thomas, I have previously worked as a manual therapist, I am now a lecturer and researcher at London South Bank University".

"Please note that this interview is solely looking to explore your own attitudes and beliefs and is no way looking to assess your medical knowledge or recollection of information from any prior clinical encounters".

1. Please tell me about your experience of low back pain.
    a) Prompt: Can you tell me about your history of LBP?
    b) Prompt: Can you tell me about your current experience of LBP?
    c) Prompt: How does your LBP affect your life?
    d) Prompt: What is your understanding of the cause of your LBP?
    e) Prompt: Can you tell me about how you see your future experience of LBP?

2. How do you feel your LBP should be managed?
    a) Prompt: Can you tell me about any experience of manual therapy i.e., "hands-on treatment" (Massage, mobilisation, manipulation provided from a healthcare provider such as physiotherapist, osteopath, chiropractor, sports therapist)?
    b) Prompt: Can you tell me about any experience of self-management (home exercises or any other independent activities)?
    c) Prompt: What is the relationship between self-management and manual therapy (in the management of LBP)?

3. Can you tell me about your views on manual therapy (for the management of LBP)?
    a) Prompt: Are there any potential benefits of manual therapy (for the management of LBP)?
    b) Prompt: What is the role of manual therapy (in the management of LBP) over the long-term?
    c) Prompt: What are your thoughts about manual therapy being necessary to manage LBP?
    d) Prompt: Are there any concerns you have about manual therapy (for the management of LBP)?

4. Can you tell me your understanding of how manual therapy works?

5. Can you tell me your views about your (working) relationship with the healthcare provider carrying out the manual therapy?

6. Is there anything else about manual therapy (for the management of LBP) you would like to tell me?
